# The History of mARC

**DOI:** 10.3390/molecules28124713

**Published:** 2023-06-12

**Authors:** Bernd Clement, Michel A. Struwe

**Affiliations:** 1 Pharmazeutisches Institut, Christian-Albrechts-Universität zu Kiel, Gutenbergstraße 76, 24118 Kiel, Germany; mstruwe@strubio.uni-kiel.de; 2 Zoologisches Institut—Strukturbiologie, Zentrum für Biochemie und Molekularbiologie, Christian-Albrechts-Universität zu Kiel, Am Botanischen Garten 1−9, 24118 Kiel, Germany

**Keywords:** mARC, molybdenum enzyme, biotransformation, reductase, prodrug

## Abstract

The mitochondrial amidoxime-reducing component (mARC) is the most recently discovered molybdoenzyme in humans after sulfite oxidase, xanthine oxidase and aldehyde oxidase. Here, the timeline of mARC’s discovery is briefly described. The story begins with investigations into *N*-oxidation of pharmaceutical drugs and model compounds. Many compounds are *N*-oxidized extensively in vitro, but it turned out that a previously unknown enzyme catalyzes the *retro*reduction of the *N*-oxygenated products in vivo. After many years, the molybdoenzyme mARC could finally be isolated and identified in 2006. mARC is an important drug-metabolizing enzyme and *N*-reduction by mARC has been exploited very successfully for prodrug strategies, that allow oral administration of otherwise poorly bioavailable therapeutic drugs. Recently, it was demonstrated that mARC is a key factor in lipid metabolism and likely involved in the pathogenesis of non-alcoholic fatty liver disease (NAFLD). The exact link between mARC and lipid metabolism is not yet fully understood. Regardless, many now consider mARC a potential drug target for the prevention or treatment of liver diseases. This article focusses on discoveries related to mammalian mARC enzymes. mARC homologues have been studied in algae, plants and bacteria. These will not be discussed extensively here.

## 1. From *N*-Oxygenation to *N*-Reduction

According to our current knowledge, mARC enzymes act as reductases. However, rather than with reduction, this story begins with oxidation. My early research career was focused on studying the metabolic biotransformation of pharmaceutical drugs. For example, during my postdoc phase at Chelsea College, London, I discovered the *N*-oxygenation as a novel biotransformation pathway for promethazine in rabbit liver [1]. During my “habilitation” in Freiburg, I was able to show that benzamidine (BA) and several ring-substituted derivatives can be oxidized to benzamidoxime (BAO) by rabbit liver homogenates [2]. This reaction appeared to be catalyzed by P450 monooxygenases rather than flavin-containing monooxygenase (FMO) [3,4], disproving the previously proposed “pK_a_” concept of *N*-oxidation [5,6], according to which nonbasic nitrogen compounds are oxidized by P450 monooxygenases, whereas basic nitrogen species are oxidized by FMO. *N*-oxygenation towards the respective amidoxime even takes place for *N*-substituted BA derivatives, such as *N*-*tert*-butyl benzamidine [7] or *N*-phenyl benzamidine [4], whereas in certain *N*-substituted BA derivatives, *N*-oxygenation can lead to subsequent *N*-dealkylation [8]. My group conducted further investigations into the oxidation of benzamidoxime and its derivatives by cytochrome P450 monooxygenases, trying to find structure–activity relationships and mechanistic insights [9]. Benzamidine and benzamidoxime have remained important model compounds in my laboratories for the past decades.

Despite otherwise being similar to amidines, *N*-oxygenation of guanidines was not observed [10]. However, many related nitrogen functions such as *N-N’*-diphenylguanidines [11], aminoguanidines [12] or the nucleobase adenine [13] could all be shown to undergo similar *N*-oxygenations as amidines. It was concluded that *N*-oxygenation by cytochrome P450 monooxygenases is possible for any nitrogen-containing functional group without α-*H* atoms [14]. In the presence of α-*H* atoms, *N*-dealkylation is the competing reaction [15].

**Figure 1:**
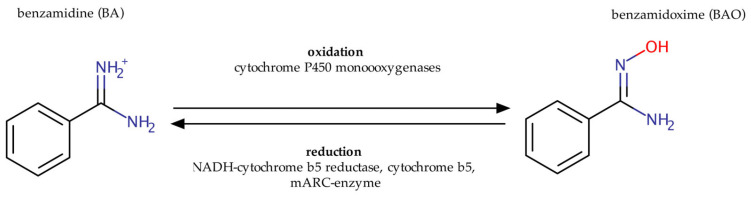
Oxidation and of benzamidine (BA) to benzamidoxime and retroreduction of benzamidoxime to benzamidine.

Oxidation of highly polar amidines such as BA by cytochrome P450 enzymes (Figure 1) was in fact a very surprising observation at the time. It had previously been believed that only hydrophobic xenobiotics would undergo phase I biotransformation (functionalization) [16] in order to increase their hydrophilicity and allow phase II biotransformation (conjugation) [17], the biological purpose being to facilitate excretion. Amidines and guanidines are, however, already very hydrophilic, and conjugation is not necessary for their elimination via the kidney. Oxidation of these compounds decreases their hydrophilicity and might therefore necessitate phase II biotransformation, despite the parent compounds being highly soluble prior to phase I biotransformation. Indeed, we could show that products obtained by *N*-oxygenation of amidines are subject to additional Phase II biotransformation reactions [18], the same being true for *N*-hydroxylated guanidines [19].

While the *N*-oxygenation of BA to BAO was evident enough in vitro, when tissue homogenate supernatants were used, I was, surprisingly and at the time very disappointingly, not able to show this oxidation to happen in vivo. The reason for this only became obvious later: while BA can be oxidized to BAO by cytochrome P450 monooxygenases, there was a second reaction happening, specifically, retroreduction of BAO to BA, which is absolutely dominant in vivo.

At the time, it was common to study biotransformation with microsomal fractions of tissue homogenates. In rabbit liver microsomes (12,000× *g* supernatant), oxidation of BA to BAO was much more pronounced than the reduction [20]. On the other hand, when 9000× *g* supernatants of other species are used, considerable reduction of BAO to BA can be detected [21]. At the same time, *N*-reduction of another benzamidoxime derivative was reported [22].

After parenteral administration of BA to rats and rabbits, only the glucuronide conjugate of BAO, but no free BAO, was detected in the animals’ urine, indicating that while *N*-oxygenation does occur, the product is not metabolically stable. However, when BAO was administered, the reduced BA was detected in high concentrations, highlighting the importance of *N*-reduction in vivo [23].

## 2. The Prodrug Principle: Amidoximes Instead of Amidines

The antiprotozoal compound pentamidine was an early example for a medicinal drug containing two amidine moieties, which can undergo *N*-oxygenation to form either a *mono*-hydroxylated or a *bis*-hydroxylated product through enzymatic *N*-oxygenation. In vitro models using *Plasmodium falciparum* or *Leishmania mexicana* showed these *N*-hydroxylated pentamidine derivatives to have practically no antiprotozoal activity, correlating with a significantly decreased DNA binding [24]. In contrast, our group had previously synthesized both the *mono*-hydroxylated and *bis*-hydroxylated pentamidine derivatives and studied their antiprotozoal activity in vivo, where both compounds were active [25]. These apparently contradicting results can be explained by reduction of the amidoxime compounds to pentamidine in vivo. Only the non-oxidized pentamidine is pharmacologically active, but hydroxylated derivatives are activated by *N*-reduction [26].

Remarkably, we detected high pentamidine concentrations in livers, lungs and kidney of rats after oral administration of *bis*-hydroxylated pentamidine, despite the nonoxygenated pentamidine not being orally bioavailable [26], implying that *N*-oxygenation can be used to increase oral bioavailability of highly polar nitrogen-containing compounds. As was shown through in vivo SPECT imaging of [**^123^**I]-labeled compounds, administration of pentamidine prodrugs might have advantages going beyond oral bioavailability, e.g., higher concentrations in the brain or different elimination pathways [27].

Amides are strongly basic (pKa ≈ 10–12), and thus carry a permanent positive charge in aqueous solution, which prohibits their diffusion across biological membranes. Amidoximes, on the other hand, are much less basic and can be absorbed in the gastrointestinal tract. The reduction of amidoximes to amidines can be exploited for oral administration of drug substances containing amidine moieties: the corresponding amidoxime is administered as a prodrug and rapidly reduced after absorption in the gastrointestinal tract [28].

**Figure 2:**
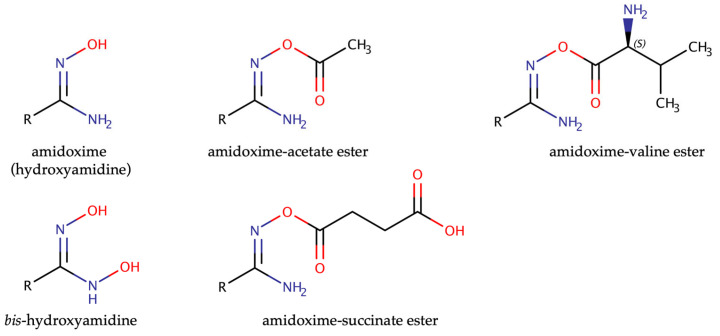
Examples for prodrug-based amidoximes.

Amidoxime prodrugs offer great flexibility, as the amidoxime functional group can be derivatized further to tune physicochemical properties (Figure 2). In the case of the model compound benzamidine, the *N,N*′-dihydroxyamidine derivative was shown to have an even higher bioavailability compared to the simple amidoxime [29]. Alternatively, the amidoxime −*OH* group can be used to form esters with various carboxylic acids such as acetic acid, succinic acid or even amino acids such as valine. After absorption, these esters are rapidly hydrolyzed by esterases, thus releasing the amidoxime, which can be reduced to the active amidine [30]. Also, the amidoxime might be incorporated into an ether-like structure, which is cleaved to the amidoxime by peptidylglycine α-amidating monooxygenase (PAM), which might allow targeting of drug substances to the central nervous system, where PAM expression levels are highest [31].

Many novel drug candidates containing amidine functional groups were developed in the 1990s as potential anticoagulants, to replace vitamin K inhibitors warfarin and phenprocoumon, which had so far been the only orally available anticoagulants. Vitamin K inhibitors are very difficult to manage in outpatient care, interact with other drugs or even foods and can have very dangerous side effects [32]. Pharmaceutical companies tried to develop alternatives, which would only inhibit selected steps within the coagulation cascade, such as factor Xa, factor IIa (thrombin) or glycoprotein IIb/IIIa [33].

**Figure 3:**
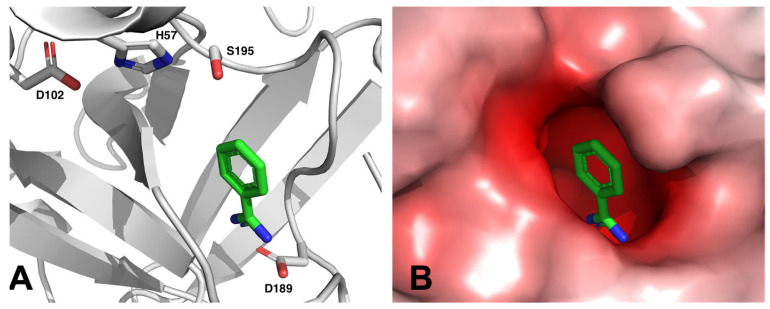
Benzamidine binds to thrombin by forming a salt bridge with aspartate 189 in proximity to the catalytic triad consisting of serine 195, histidine 57 and aspartate 102, as can be seen in the crystal structure of benzamidine-bound human thrombin (PDB: 4UEH) [36]. Panel (**A**): Cartoon representation. Panel (**B**): Electrostatic surface representation. The electrostatic potentials were calculated with APBS [37] and are represented as a color gradient from red—10 k_B_T/e_c_ over white 0 k_B_T/e_c_ to blue +10 k_B_T/e_c_.

Many of these drug candidates were derivatives of BA [34]. Most targets within the coagulation cascade are serine proteases, and BA was known as an inhibitor for serine proteases for a long time [35]. Benzamidine can bind into a negatively charged pocket next to the active site of these serine proteases, where it mimics an arginine side chain of the protease’s substrate and forms a salt bridge to a conserved aspartate residue (see Figure 3).

The BA moiety imitating the guanidine functional group of arginine in the natural substrate fibrinogen is therefore strictly required for the pharmacokinetic properties of these novel anticoagulant drugs and cannot be replaced by a less polar group. However, the free amidines usually lead to poor bioavailability. In many cases, the amidoxime prodrug principle can be applied successfully to increase the oral bioavailability, e.g., for the glycoprotein IIb/IIIa antagonist lamifiban [38] or sibrafiban [39], as well as factor Xa inhibitors [40,41].

Because of our patent and publications on the prodrug strategy “amidoximes instead of amidines”, we were approached by Astra Zeneca. A common project started to identify the enzyme responsible for reduction of amidoximes to amidines such as ximelagatran to melagatran (vide infra).

**Figure 4:**
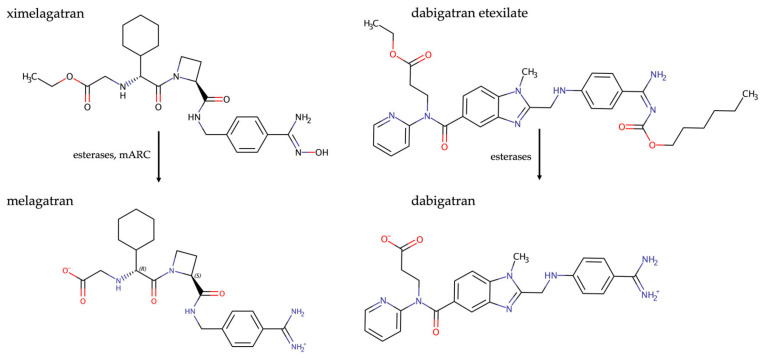
Prodrug principles of ximelagatran and dabigatran etexilate.

Of particular note is the factor IIa inhibitor (thrombin inhibitor) melagatran, where the amidoxime prodrug ximelagatran had an oral bioavailability of 18–24% compared to 3–7% for the active compound [42,43]. Ximelagatran was an important milestone, as it was approved in 2003 as the first of the “new oral anticoagulants” (NOACs). Unfortunately, a small portion of patients treated with ximelagatran showed transiently increased asymptomatic liver transaminase levels [44], which eventually led to a complete market withdrawal in 2006 [45]. Another factor II inhibitor, dabigatran, remains available. Like melagatran, dabigatran is a BA derivative (Figure 4) and is not orally bioavailable in its active form. Instead of using an amidoxime prodrug approach, the amidine was incorporated into a urethane group, which releases the active amidine after absorption by enzymatic hydrolysis [46]. However, it should be noted that with this strategy, oral bioavailability of the prodrug dabigatran etexilate remains quite low (approx. 7%) [47], even though an elaborate formulation was developed, in which dabigatran etexilate is coated into tartaric acid pellets to provide a microacidic absorption environment [48]. We could demonstrate that the amidoxime prodrug approach works just as well for this amidine [49].

The amidoxime prodrug principle was shown to work for a series of other amidines. Examples are the urokinase inhibitor upamostat [50], which has recently been studied as a potential treatment against SARS-CoV-2 [51], investigational antiviral drugs [52,53] or novel nitric oxide synthase inhibitors [54].

Modern drug development pipelines can produce drug candidates with practically any functional groups. Prodrugs based on amidoximes remain an interesting approach for more favorable physicochemical and pharmacokinetic properties.

## 3. Discovery of the mARC Enzyme System

While there had been plenty of evidence that the oxidation of amidines towards amidoximes is catalyzed by cytochrome P450 monooxygenases, we did not know which particular enzymes were responsible for the reduction of amidoximes to amidines.

In fact, many other authors had previously studied metabolic reduction of *N*-hydroxylated compounds. For example, Kadlubar and Ziegler identified an “NADH-Dependent *N*-Hydroxy Amine Reductase” in pig liver microsomes. It became clear that *N*-reduction was catalyzed by a complex of at least three proteins: cytochrome b5 (CYB5), its flavin-containing reductase and a third, unidentified component. This unknown third protein had no strongly absorbing cofactors and was insensitive to oxygen or typical cytochrome P450 inhibitors [55,56]. Similarly, *N*-reduction had previously been observed in rat liver mitochondria [57,58].

Over the years, many attempts were made to solve the “mystery of the third protein”, which catalyzes *N*-reduction together with cytochrome b5 and NADH-cytochrome b5 reductase (NB5R). There were different potential candidates, such as a cytochrome P450 subfamily 2D member [59], or stearoyl-CoA desaturase [60,61], which are capable of reducing amidoximes to amidines, but with very low conversion rates. Thus, it seemed that these enzymes would not be responsible for the extensive in vivo reduction.

When we investigated reduction of amidoximes in different subcellular fractions, we found that the *N*-reductive activity was significantly higher in mitochondria compared to microsomes. Furthermore, it could be shown that *N*-reductive activity was strongly enriched in preparations of the outer mitochondrial membrane (OMM).

Eventually, my PhD student Antje Havemeyer was able to discover the “third protein” in 2006. Through a rigorously improved purification protocol, she was able to finally isolate the third component of the mitochondrial amidoxime-reducing complex and identify it by mass spectrometric techniques. It was furthermore discovered that our newly found “mitochondrial amidoxime reducing component” (mARC) contained molybdenum cofactor (Moco) and is homologous to molybdenum cofactor sulfurase [62]. All mammalian genomes encode two paralogues of mARC, mARC1 and mARC2. Prior to the discovery of mARC, the genes were called *MOSC1* and *MOSC2*, as they belong to a diverse superfamily of proteins referred to as “MOSC domain proteins” [63]. The gene names were accordingly changed to *MARC1* and *MARC2*. Recently, the HUGO Gene Nomenclature Committee decided to change the gene names to *MTARC1* and *MTARC2*, as *MARC1* and *MARC2* are commonly misinterpreted as dates (1 March/2 March) by Microsoft Office Excel (Microsoft, Redmond, WA, USA) [64].

At this point, I had been a medicinal chemist with a focus on drug metabolism and did not know much about molybdenum and its biochemistry. We therefore went looking for collaborators with expertise in these matters and got in touch with Ralf Mendel and Florian Bittner, who had been studying molybdenum enzymes and the molybdenum cofactor biosynthesis at TU Braunschweig for a long time [65]. They introduced me to the molybdenum community, and I am truly grateful for the productive collaboration that my group maintained with Ralf and Florian for many years.

Together with Ralf and Florian, we were able to prepare mARC enzymes recombinantly and prove their *N*-reductive activity in reconstituted in vitro HPLC-based activity assays [66,67]. Over the years, alternative mARC activity assays have been developed, e.g., an assay based on photometric measurement of NADH consumption [68] or different electrochemical systems [69,70], developed in collaboration with the group of Paul Bernhardt in Brisbane.

**Figure 5:**
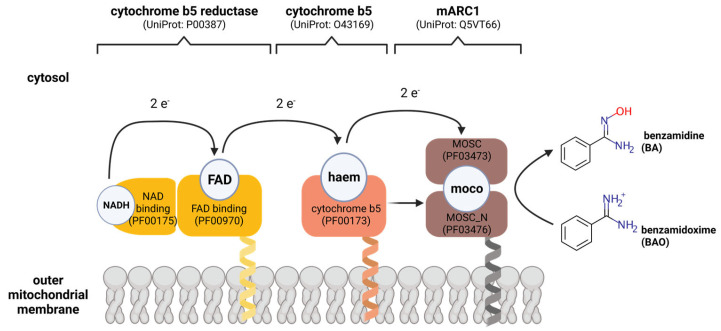
Putative electron transport chain of the mammalian mARC enzyme system. Figure created with *BioRender*.

It was confirmed that mARC is localized in the OMM [71], and ***N***-reductive activity requires the mitochondrial isoform of CYB5 (CYB5B), not the microsomal isoform (CYB5A) [72], and that the relevant isoform of NB5R is NB5R3 [73]. Figure 5 depicts the putative electron flow from NADH to an *N*-oxygenated product via CYB5, NB5R and mARC.

A great milestone in recent mARC research was the successful determination of the mARC1 crystal structure in 2018 [74]. At that point, I had already started a very fruitful collaboration with the Axel Scheidig group at Kiel University’s structural biology department, but the mARC enzyme proved very difficult to crystallize. Eventually, the structure could be determined using an internal fusion protein approach with T4 lysozyme [75]. The extensive and successful collaboration is ongoing unabated.

## 4. Substrates of mARC

While mARC enzymes were initially discovered through the reduction of benzamidoxime, many other substrates have been studied over the years. We have tested a great variety of substituted benzamidoxime derivatives [76] and were unable to spot any clear structure–activity relationships. mARC also reduces *N*-hydroxyguanidines such as *N*^ω^-hydroxy-l-arginine [77] or guanoxabenz [78], *N*-hydroxy sulfonamides such as *N*-hydroxy-benzenesulfonamide or *N*-hydroxy-valdecoxib [79] and *N*-hydroxamic acids such as benzhydroxamic acid, vorinostat or bufexamac [80]. *N*-oxides such as nicotinamide *N*-oxide [78] can also be reduced; even trimethylamine *N*-oxide, albeit with very low turnover rates [81].

**Figure 6:**
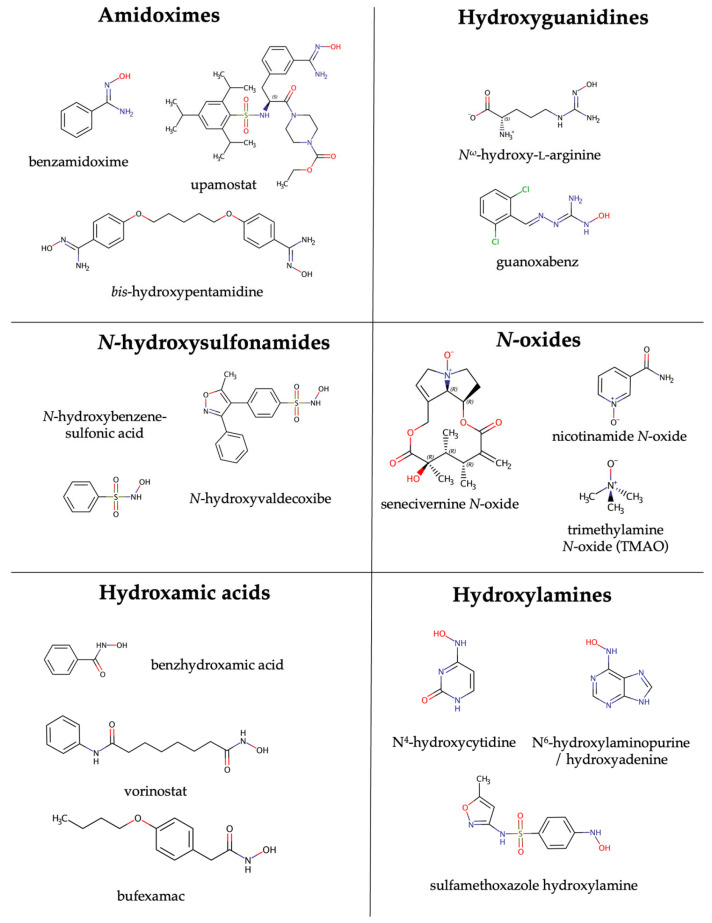
Some examples for mARC substrates.

In drug metabolism, much attention is usually paid towards oxidations, but reductions, like those catalyzed by mARC enzymes, should also be considered. Particularly for nitrogen-containing functional groups, reductions are as important as oxidations [82]. mARC is a drug-metabolizing enzyme, as it can activate *N*-hydroxylated prodrugs (vide supra). However, mARC can also inactivate drug substances when they rely on functional groups incorporating *N-OH* bonds. For example, hydroxamic acids can be used in pharmacophores targeting metalloproteins. This concept is used for inhibitors of zinc-containing histone deacetylase [83], or the matrix metalloproteinase-13 inhibitor CP-544439. Some examples for known mARC substrates are shown in Figure 6.

Similarly, the cytostatic agent *N*-hydroxyurea, an inhibitor of ribonucleotide reductase, is used in sickle-cell disease and certain types of cancer and is an excellent substrate of mARC1 [68], which is likely the reason for its very short half-life time [84].

Another aspect worth considering is the detoxification reactions catalyzed by mARC. Shortly after our discovery of mammalian mARC enzymes, Kozmin et al. discovered that two mARC homologues, YiiM and YcbX, contribute towards resistance of *Escherichia coli* bacteria towards 6-hydroxylaminopurine [85]. Similarly, mARC enzymes have been shown to reduce mutagenic *N*-hydroxylated nucleobases and nucleotides [86] and protect cells against their harmful effects [87]. Toxic *N*-hydroxylated metabolites are often formed in vivo. For example, sulfamethoxazole is oxidized in vivo to a toxic hydroxylamine, which can be detoxified by mARC [88].

The analgesic drug phenacetin is metabolically oxidized to the hydroxamic acid *N*-hydroxyphenacetine, a strongly mutagenic compound held responsible for phenacetin’s severe side effects [89], which have led to its withdrawal from the market. *N*-hydroxyphenacetine is not reduced by mARC [80]. We have long hypothesized that any exogenous compounds possessing nitrogen groups that can undergo metabolic oxidation are potentially toxic, unless they can be detoxified by mARC or another mechanism.

It should be emphasized though that *N*-reduction by mARC can also, in very specific cases, lead to toxification. Pyrrolizidine alkaloids are used by many plants as a defense against herbivores. These alkaloids are inactive as *N*-oxides but highly toxic in their amine form. We could show that senecivernine *N*-oxide can be reduced by mARC to release the toxic senecivernine amine. In this case, oxidation, i.e., by flavin-containing monooxygenases or cytochrome P450 enzymes, would present a detoxification, but reduction by mARC a toxification [68].

Whether or not any of the functional groups we have shown to be reduced by mARC have any connection to the enzymes’ physiological function is currently unknown. It has been hypothesized that mARC might play a role in nitric oxide homeostasis, either by reducing the nitric oxide precursor [77] or by acting as a nitrite-dependent nitric oxide synthase, i.e., catalyzing a one-electron reduction of nitrite to nitric oxide [90]. Whether this reaction is relevant under physiological conditions remains unclear, as it appears to occur only under anaerobic conditions [90,91].

## 5. mARC Enzymes in Human Disease

So far, we have mostly looked at mARC as an enzyme involved in metabolism of xenobiotics. More and more, however, it appears that mARC enzymes might have functions related to physiological processes and are involved in human disease.

One very interesting case is the involvement of mARC in hepatocellular carcinoma (HCC). It was found that mARC2 could suppress the progression of HCC, due to competition with the tumor suppressor protein p27 for degradation by the same ubiquitin E3 ligase RNF123 [92]. We find this mechanism very interesting as it is presumably completely unrelated to mARC2’s enzymatic activity and therefore represents a true moonlighting function. Other authors have previously suggested that mARC proteins might be moonlighting proteins fulfilling multiple physiological functions [93]. A follow-up study found a negative correlation between expression levels of *MTARC2*, *CYB5* and *NB5R* with HCC tumor size, progression and risk of metastasis. It was suggested that expression levels of ***MTARC2*** and its electron carrier proteins could serve as a prognostic marker in patients with HCC [94].

Right now, the big story, however, is the involvement of mARC1 in liver disease, more specifically in non-alcoholic fatty liver disease (NAFLD) and non-alcoholic steatohepatitis (NASH). In 2020, Emdin et al. published a genome-wide association study (GWAS) in which the common p.A165T variant of human mARC1 appeared to convey a protective effect against liver cirrhosis, decrease liver fat, circulating liver enzymes and blood cholesterol levels [95]. An involvement of mARC enzymes in lipogenesis had been described previously [96,97]. *MTARC2* knockout mice possess a very distinctive phenotype: The animals are resistant to body weight gain induced by a high-fat diet and have lower blood cholesterol levels [98,99], proving that mARC has a crucial function in lipid metabolism.

Nonetheless, the results of Emdin et al. regarding the mARC1 variant came as a surprise to us, as we had studied the influence of various common mutations on the in vitro activities and molybdenum contents of mARC enzymes years earlier. In this study, the mARC1 p.A165T variant did not have altered catalytic properties [16]. The mutated residue A165 is some 25 Å away from the active site of mARC1, and despite some in silico studies suggesting that the variant protein would “cause loss of the alpha-helix and alter the metal-binding ability of MTARC1” as well as “affect the overall stability of the protein” [100], our own X-ray crystallographic analysis of the variant protein revealed no structural differences between the wild type and variant [101]. The mechanism by which the mARC1 p.A165T variant influences liver disease remains unknown, and it might require significant efforts to understand it [102].

Notwithstanding, the connection between mARC1 and liver disease has been confirmed by numerous other GWAS [100,103,104,105,106,107,108]. NAFLD is a very prevalent disease; the number of patients affected by NAFLD in the United States alone is expected to exceed 100 million by 2030, with 27% of these cases progressing to NASH. There are, as of yet, no pharmacotherapeutic options for the treatment and prevention of NAFLD and NASH [109].

mARC1 might represent an interesting drug target, especially in obese patients with a high risk of liver mortality [103,108]. Consequently, many pharmaceutical companies are currently investigating *si*-RNA-based approaches for targeted reduction of mARC1 expression levels in the liver (see Table 1).

## 6. Outlook

Over the course of the years, our view on mARC enzymes has changed significantly. In the beginning, the enzymes were mostly of interest with relation to the activation of amidoxime prodrugs. As we got to learn more about mARC, we viewed it more as a metabolic enzyme involved in biotransformation reactions of xenobiotics. More recently, the focus has shifted towards the involvement of mARC enzymes in human disease, particularly, NAFLD and NASH. It will be exciting to learn more about the role of mARC in disease and whether or not mARC, in fact, will one day become a target for therapeutic drugs. Table 2 gives a very brief summary of the most important milestones in the story of human mARC enzymes.

## 7. Summary

As usual, our research on mARC had ups and downs. Very disappointing was the withdrawal of ximelagatran using our prodrug principle. Although the side effects are not due to the reduction by mARC, our prodrug strategy consequently became less popular. On the other hand, this prodrug principle was the starting point for the discovery of mARC, which turned out to be a general drug-metabolizing enzyme and just recently also turned out to be a potential drug target. While this article focused on the role of mammalian mARC enzymes, a lot of important work has been conducted with respect to homologues of these proteins from algae, plants and bacteria. We encourage readers to also study review papers that put a stronger emphasis on these enzymes [93,112].

## Figures and Tables

**Table 1:** Recent patents regarding mARC1 as a target for the prevention and/or treatment of liver disease.

Patent Number	Company	Title
WO2023282704	OliX Pharmaceuticals	Asymmetric siRNA targeting MARC1 gene, and use thereof
WO2022248665	Novo Nordisk	Compositions and methods for inhibiting mitochondria amidoxime-reducing component 1 (MARC1) expression
WO2022183065	Ionis Pharmaceuticals	Modulation of MARC1 expression using antisense oligonucleotides or other inhibitors to treat liver disease
WO2022159158	Alnylam Pharmaceuticals	Modified double-stranded oligonucleotides
WO2022036126	Amgen	RNAi constructs and methods for inhibiting mARC1 expression
WO2021237097	Alnylam Pharmaceuticals	Compositions and methods for inhibiting MARC1 gene expression in human using double-stranded RNA
US20210262022	The General Hospital Corporation	Mitochondrial amidoxime-reducing component (MARC) gene variants associated with liver diseases and methods of protection against liver diseases or symptoms thereof
WO2020154567	Viscient Biosciences	Compositions and methods for the diagnosis and treatment of diseases of the liver

**Table 2:** Important milestones in the story of human mARC enzymes.

Year	Discovery	Citation
1983	BA is oxidized to BAO in vitro	[2]
1988	*N*-reduction of BAO to BA is demonstrated in vitro	[21]
1993	In vivo studies show that N-reduction of BAO dominates physiologically	[23]
2003	The mARC-activated prodrug Ximelagatran is submitted to the FDA for approval	[110]
2005	*N*-reducing activity is highest in mitochondria	[111]
2006	mARC proteins were first isolated from porcine liver	[62]
2010	Detailed description of recombinant human mARC proteins	[67]
2017	Initial development of an electrochemical assay for mARC activity	[69]
2018	Crystal structure of human mARC1	[74,75]
2019	In vivo murine *MTARC2* knockout model underscores mARC’s role in lipid metabolism	[98]
2019	Development of a fast spectrophotometric mARC activity assay	[68]
2020	The mARC1 p.A165T variant is first shown to protect against liver disease	[95]

## Data Availability

Not applicable.
